# Cholesterol and prostate cancer risk: a long-term prospective cohort study

**DOI:** 10.1186/s12885-016-2691-5

**Published:** 2016-08-17

**Authors:** Trond Heir, Ragnhild Sørum Falk, Trude Eid Robsahm, Leiv Sandvik, Jan Erikssen, Steinar Tretli

**Affiliations:** 1 Oslo Ischemia Study, Oslo University Hospital, N-0407 Oslo, Norway; 2 Oslo Centre for Biostatistics and Epidemiology, Oslo University Hospital, Oslo, Norway; 3 Department of Research, Cancer Registry of Norway, Oslo, Norway

## Abstract

**Background:**

Few studies have taken risk of competing events into account when examining the relationship between cholesterol and prostate cancer incidence, and few studies have a follow-up over several decades. We aimed to use these approaches to examine the relationship between cholesterol and prostate cancer.

**Methods:**

A cohort of 1997 healthy Norwegian men aged 40–59 years in 1972–75 was followed throughout 2012. Cancer data were extracted from the Cancer Registry of Norway. The association between cholesterol and prostate cancer incidence was assessed using competing risk regression analysis, with adjustment for potential confounders. Date and cause of death was obtained from the Cause of Death Registry of Norway.

**Results:**

The study cohort had a cancer risk similar to the general Norwegian population. Prostate cancer was registered in 213 men (11 %), including 62 (3 %) with advanced stage at diagnosis. For overall and advanced stage prostate cancer, the incidence was twice as high in the lowest quartile of cholesterol compared to the highest quartile. These associations remained significant after adjustment for age, smoking, physical fitness, BMI, and systolic blood pressure. Furthermore, high physical fitness and low BMI were associated with increased prostate cancer incidence. Sensitivity analyses excluding events during the first 20 years of observation revealed similar results.

**Conclusion:**

Low cholesterol, as well as high physical fitness and low BMI, may be associated with increased risk of prostate cancer. These findings conflict with current prostate cancer prevention recommendations.

**Electronic supplementary material:**

The online version of this article (doi:10.1186/s12885-016-2691-5) contains supplementary material, which is available to authorized users.

## Background

The role of cholesterol and prostate cancer has been a topic of research for many years. Several observational studies in the 1980s aroused suspicion of an association between low cholesterol and increased prostate cancer risk [1–3]. With notable exceptions [4–7], a majority of recent studies have pointed to a positive association between total cholesterol and overall prostate cancer incidence [8–12], or between cholesterol and high-grade prostate cancer [9, 11–14]. In addition, prostate cancer mortality has been reported to be higher among those with initial high cholesterol levels [15], although this finding is not consistent [16].

From a cell biological point of view, cholesterol may play an important role in prostate cancer cell growth [17]. A review of a series of reports suggests that use of statins in cholesterol-lowering therapies may reduce the risk of aggressive prostate cancer [18]. A possible implication is that cholesterol-lowering therapies may provide an opportunity to alter the disease course [19].

However, the role of cholesterol in prostate cancer is still a challenging research question. Firstly, studies have not consistently confirmed an association between total cholesterol and overall or high-grade prostate cancer incidence [4–7]. Secondly, cholesterol is a well-known risk factor of cardiovascular disease and mortality [20]. Few studies to date have taken competing events into account when examining risk of prostate cancer. Using conventional methods such as the Cox model, deaths are censored, despite the fact that they may be considered competing events in the analysis of prostate cancer. The possibility that this may affect the conclusion of a study has been demonstrated by Haggström and colleagues. In a large longitudinal study that in the first case of Cox regression analysis revealed no associations between metabolic aberrations and prostate cancer risk [21], they found that men with metabolic aberrations had lower risk of prostate cancer when competing events were taken into account [22]. This calls for more studies assessing the association between cholesterol and prostate cancer, taking risk of competing events into account.

Thirdly, the tumor cell proliferation may affect the circulating cholesterol level due to the high metabolic rate of cancer cells that requires cholesterol esterification [17]. Thus, a cholesterol-lowering effect of cancer itself may result in an excess of pre-existing cancer among individuals with low cholesterol levels. Multiple cholesterol measures over time have revealed that in some individuals cholesterol levels may be lower than expected at as much as 16–18 years before diagnosis, while in others cholesterol levels decline close to the time of cancer diagnosis [23]. This emphasizes the need of restricting analyses to healthy subjects, and examining results by excluding those who received a cancer diagnosis through a sufficiently long initial period of follow-up.

Lastly, screening procedures for prostate cancer may have influenced incidence rates during the last two decades and especially increased the proportion of non-aggressive tumors. The possibility of over-diagnosis actualizes the need to pay special attention to advanced stage cancer or prostate cancer-specific deaths.

Based on a cohort of initially healthy middle-aged men followed for 40 years [24–26], we aimed to examine the relationship between total cholesterol and the incidence of overall and advanced stage prostate cancer, focusing on the challenges mentioned above.

## Methods

### Data sources

The Oslo Ischemia Study; a cohort of working males, aged 40 to 59 years, recruited from five companies in Oslo, Norway, in the period 1972–75. They were apparently healthy, free from somatic diseases and not using drugs. Details about the selection criteria are presented elsewhere [26, 27]. In total, 2341 employees were invited, of whom 2014 (86 %) participated by completing the study protocol.

Information on cancer and cause of death were obtained through linkages between the cohort data, the Cancer Registry of Norway and the Cause of Death Registry, respectively. The data were linked through the unique national identity numbers assigned to every individual residing in Norway from 1960 onwards. The Cancer Registry of Norway has registered all cancer diagnoses nationwide from 1953 onwards. Mandatory reporting from multiple independent sources ensures the collection of complete and high quality data [28]. The Cause of Death Registry contains information on all recorded deaths of Norwegian citizens living in Norway at time of death since 1951.

### Measurements

In accordance with the study protocol, a comprehensive medical history was taken from all participants in addition to an extensive physical examination, a chest X-ray, a panel of fasting blood tests, and a maximal exercise tolerance bicycle test. All participants were asked to abstain from smoking for at least 8 h, and eating for 12 h, in advance of the examination.

Total serum cholesterol was analyzed by Liebermann Burchart's no-enzymatic method [29] and divided into quartiles. Smoking status was categorized as never or ever smoker. Physical fitness was measured as work capacity (sum of work performed in the bicycle test) divided by body weight, kJ/kg, and divided into tertiles. Body mass index (BMI) was calculated based on objectively measured height and weight (body weight/height^2^, kg/m^2^), and divided into normal weight (BMI < 25) or overweight (BMI ≥ 25). Resting blood pressure was measured after the participant had been in the supine position for five minutes [30], and systolic blood pressure was divided into quartiles. Participants were encouraged to continue exercising until exhaustion [24]. Age at inclusion was divided into four groups (<45, 45–49, 50–54, 55+ years).

The cancer data consisted of information on cancer type (according to the International Classification of Disease, revision 7), date of diagnosis and stage of disease at diagnosis. Stage at diagnosis was divided into two categorises based on information on metastases at diagnosis: localized (invasive prostate cancer without any metastases) and advanced (any infiltration into surrounding structures, regional or distant metastases). Cases lacking information on metastasis were categorized as “unknown”.

The cohort of men was followed throughout 2012. Of the 2014 men included, we excluded two men with missing vital status data and 15 men due to cancer diagnosis prior to date of the examination. Thus, 1997 men remained in the cohort for analyses of prostate cancer incidence. Of these, 1520 men were followed for 20 years or more. The registrations of baseline variables used in this study were 100 % complete for all men included.

### Statistical analyses

The baseline characteristics are presented as frequencies and proportions. Comparisons of proportions were tested by chi-square tests.

Standardized incidence rates (SIR) were computed to measure the relative risk of cancer (overall and the six most prevalent sites) in the cohort compared to the general male population in the two counties (Oslo and Akershus) where the men were recruited. Cancer incidences were extracted from the Cancer Registry of Norway. Reference rates were computed for 5-year age groups and 3-year calendar periods. Expected numbers were computed by applying the age- and period-specific rates to the observed person-years in the cohort. SIRs were computed by taking the ratio of the observed to expected incidence, with accompanying 95 % confidence intervals (CI).

The cohort of men was followed up longitudinally from the date of examination to the date of diagnosis of prostate cancer. Cumulative incidence of prostate cancer was estimated in competing risk models, where death (all causes) was considered as a competing event. The men were censored at the date of emigration or study end (December 31, 2012). Cumulative incidence of prostate cancer, which describes the absolute risk over the time course, was plotted for each quartile of cholesterol level. Quartile 1 was compared to quartile 4 by performing the Pepe and Mori test of equality in cumulative incidences [31].

To evaluate the effect of cholesterol on prostate cancer incidence, we performed competing risk regression analysis with adjustment for potential confounding factors; age, smoking, physical fitness, BMI, and systolic blood pressure (all categorical as given above). We used the Fine and Gray model [32], which extends the Cox proportional hazards model to competing risks data by considering the sub-distribution hazard while adjusting for the competing risk of (all cause) death. The strength of the association between each variable and the outcome was assessed using the sub-hazard ratios (SHR). Tests for trend across categories were performed by entering categorical variables as continuous in the models. The model assumptions were found to be adequately met and no significant interactions were observed.

We conducted sensitivity analyses restricted to men who were still alive and free from prostate-cancer at 20 years of follow-up, and followed them for the next 20 years. Further, we performed separate analyses for localized and advanced stage prostate cancer. In the stage-specific analysis, diagnosis at the other stage was defined as a competing event (i.e., localized stage prostate cancer was considered a competing event when studying advanced stage prostate cancer). For comparison, we conducted Cox regression analysis equivalent to the competing risk regression analysis of prostate cancer incidence. Results from the Cox analyses are presented as supplementary files.

Among men diagnosed with prostate cancer, we performed a separate competing risk analysis to evaluate the effect of cholesterol on prostate cancer death. The method of Fine and Gray was used to analyze time to prostate cancer specific death with death from other causes as a competing event.

All statistical analyses were performed using Stata [33]. In particular, we used the *stcrreg* and *stcompet* modules when analyzing competing events.

## Results

**Fig. 1:**
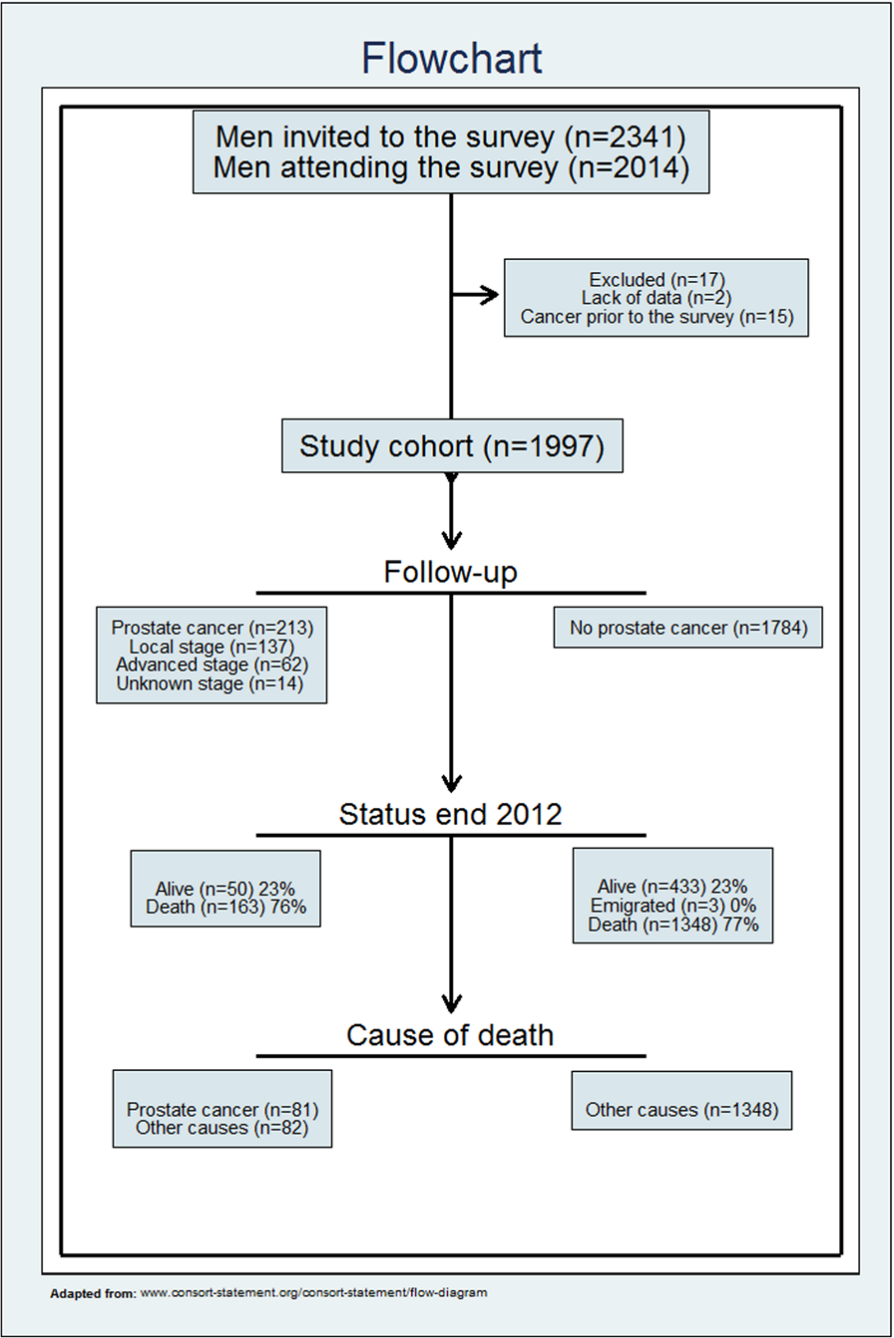
Flowchart of the cohort of initially healthy 40 to 59 year old men recruited in the period 1972–75 and followed for 40 years, Oslo Ischemia Study

During the 40 years observation period 1511 men died (all causes) and 758 were diagnosed with cancer. Prostate cancer was registered in 213 men, of which 137 were localized, 62 advanced and 14 with an unknown stage at time of diagnosis (Fig. 1). The cancer risk in the study cohort was not different from the cancer risk in the general population (Table 1). Higher cholesterol was associated with higher age, lower fitness, and higher blood pressure (Table 2). Smoking prevalence and BMI were not significantly associated with cholesterol level.

**Table 1:** Standardized incidence rates (SIR) analysis with 95 % confidence intervals (CI) for cancer (overall and the six most prevalent sites) in the cohort compared to the general male population in Oslo and Akershus

	Observed	Expected	SIR (95 % CI)
Cancer, overall	758	798.3	0.95 (0.88–1.02)
Colon	87	81.9	1.06 (0.86–1.31)
Rectum	44	47.4	0.93 (0.69–1.25)
Lung	109	126.0	0.86 (0.72–1.04)
Prostate	213	201.7	1.06 (0.92–1.21)
Bladder and kidney	104	100.7	1.03 (0.85–1.25)
Melanoma	94	83.7	1.12 (0.92–1.37)

**Table 2:** Baseline characteristics stratified by quartiles of cholesterol (numbers and proportions) in the cohort of healthy middle-aged men, *n* = 1997

		Cholesterol (mmol/l)					
		Total	Q1 (2.7–5.8)	Q2 (5.9–6.6)	Q3 (6.7–7.4)	Q4 (7.5–15.4)	*P-value*
Age at inclusion (years)	<45	469 (23.5)	155 (29.0)	130 (24.9)	103 (21.8)	81 (17.3)	<0.001
	45–49	568 (28.5)	154 (28.8)	158 (30.3)	127 (26.9)	129 (27.5)	
	50–54	550 (27.5)	126 (23.6)	147 (28.2)	139 (29.5)	138 (29.4)	
	55+	410 (20.5)	99 (18.5)	87 (16.7)	103 (21.8)	121 (25.8)	
Smoking	Ever	1495 (74.9)	386 (72.3)	389 (74.5)	351 (74.4)	369 (78.7)	0.13
	Never	502 (25.1)	148 (27.7)	133 (25.5)	121 (25.6)	100 (21.3)	
Physical fitness (kJ/kg)	T1 (21.1–118.6)	666 (33.4)	148 (27.7)	172 (33.0)	165 (35.0)	181 (38.6)	<0.001
	T2 (118.7–161.2)	666 (33.4)	164 (30.7)	163 (31.2)	161 (34.1)	178 (38.0)	
	T3 (161.3–514.9)	665 (33.2)	222 (41.6)	187 (35.8)	146 (30.9)	110 (23.5)	
Body mass index (kg/m^2^)	<25	1209 (60.5)	335 (62.7)	324 (62.1)	286 (60.6)	264 (56.3)	0.16
	≥25	788 (39.5)	199 (27.3)	198 (37.9)	186 (39.4)	205 (43.7)	
Systolic blood pressure (mmHg)	Q1 (88–118)	578 (28.9)	181 (33.9)	166 (31.8)	132 (28.0)	99 (21.1)	<0.001
	Q2 (120–128)	484 (24.2)	150 (28.1)	122 (23.4)	96 (20.3)	116 (24.7)	
	Q3 (130–140)	457 (22.9)	99 (18.5)	119 (22.8)	114 (24.2)	125 (26.7)	
	Q4 (142–220)	478 (23.9)	104 (19.5)	115 (22.0)	130 (27.5)	129 (27.5)	

T1 - T3: tertiles 1 to 3, Q1 - Q4: quartiles 1 to 4

**Fig. 2:**
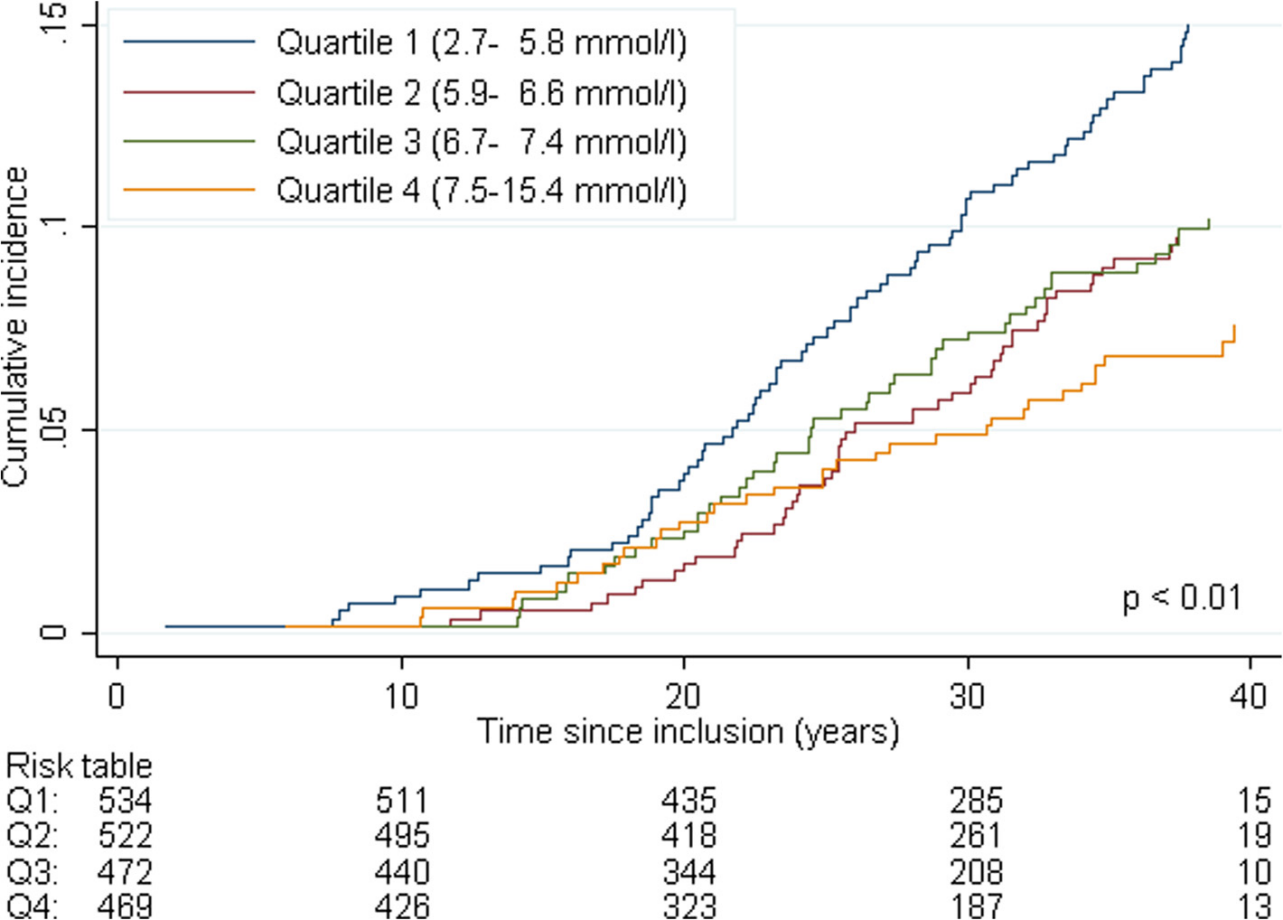
Cumulative incidence of prostate cancer by quartiles of cholesterol in a cohort of initially healthy middle-aged men followed for 40 years, *n* = 1997. The number of prostate cancer diagnosis in each quartile of cholesterol was 80 (quartile 1), 51 (quartile 2), 48 (quartile 3) and 34 (quartile 4). *P*-value achieved from the Pepe and Mori test comparing the cumulative incidence of cholesterol quartile 1 versus quartile 4

When using competing risk regression to study the association between cholesterol and risk of prostate cancer (Table 3) the incidence rate was found to be significantly higher in cholesterol quartile 1 compared to quartile 4 (SHR 2.2, 95 % CI 1.4 – 3.2). This difference remained significant when adjusting for age, smoking, physical fitness, BMI, and systolic blood pressure. In addition, the incidence rate was found to be significantly higher among men who had the highest physical fitness level (tertile 3) compared to men in the lowest level (tertile 1) (SHR 1.7, 95 % CI 1.1-2.5), and lower in men with BMI ≥25 compared to men with BMI <25 (SHR 0.69, 95 % CI 0.52–0.93) (Table 3). The effects of the covariates on the cause-specific hazard from the Cox analysis were of the same magnitude (Additional file 1: Table S1). Figure 2 shows the variation in cumulative incidence of prostate cancer by quartiles of cholesterol. Compared to quartile 4, men in quartile 1 had significantly higher risk of prostate cancer after 40 years of follow-up.

**Table 3:** Competing risk regression to evaluate the effect of selected covariates on prostate cancer incidence

	Full-time follow-up period, *n* = 1997				Restricted to more than 20 year of follow-up, *n* = 1520	
	Univariate analyses		Multivariate analysis		Multivariate analysis	
	SHR (95 % CI)	*P-value*	SHR (95 % CI)	*P-value*	SHR (95 % CI)	*P-value*
Cholesterol (mmol/l)		<0.001*		<0.001*		0.01*
Q1 (2.7 – 5.8)	2.15 (1.44–3.22)	<0.001	2.00 (1.32–3.03)	0.001	2.00 (1.20–3.34)	<0.01
(5.9 – 6.6)	1.36 (0.88–2.09)	0.17	1.29 (0.83–2.00)	0.26	1.46 (0.86–2.48)	0.16
Q3 (6.7 – 7.4)	1.42 (0.92–2.21)	0.12	1.36 (0.87–2.12)	0.17	1.55 (0.90–2.66)	0.11
Q4 (7.5 – 15.4)	1		1		1	
Age at inclusion (years)		0.48*		0.40*		0.36*
< 45	1		1		1	
45–49	0.84 (0.58–1.20)	0.33	0.92 (0.64–1.32)	0.66	0.97 (0.66–1.43)	0.89
50–54	0.87 (0.60–1.25)	0.45	1.09 (0.73–1.62)	0.67	0.86 (0.54–1.35)	0.51
55+	0.85 (0.57–1.27)	0.43	1.15 (0.75–1.79)	0.52	0.80 (0.46–1.40)	0.44
Smoking						
Never	1		1		1	
Ever	0.75 (0.56–1.01)	0.06	0.79 (0.59–1.07)	0.12	0.81 (0.58–1.14)	0.22
Physical fitness (kJ/kg)		<0.001*		0.01*		0.12*
T1 (21.1 – 118.6)	1		1		1	
T2 (118.7 – 161.2)	1.38 (0.96–1.98)	0.08	1.43 (0.98–2.07)	0.06	1.27 (0.82–1.99)	0.29
T3 (161.3 – 515.9)	1.76 (1.25–2.48)	<0.01	1.68 (1.14–2.47)	0.01	1.43 (0.92–2.22)	0.11
Body mass index (kg/m^2^)						
<25	1		1		1	
≥ 25	0.66 (0.49–0.89)	<0.01	0.69 (0.52–0.93)	0.01	0.62 (0.44–0.88)	<0.01
Systolic blood pressure (mmHg)		0.59*		0.55*		0.41*
Q1 (88 – 118)	1		1		1	
Q2 (120 – 128)	0.85 (0.59–1.23)	0.39	0.89 (0.62–1.28)	0.53	1.00 (0.66–1.51)	0.99
Q3 (130 – 140)	0.83 (0.57–1.22)	0.35	0.96 (0.65–1.40)	0.83	1.06 (0.68–1.65)	0.79
Q4 (142 – 220)	0.92 (0.64–1.32)	0.63	1.13 (0.78–1.65)	0.52	1.22 (0.78–1.93)	0.37

*SHR* sub-distribution hazard ratios, *CI* confidence interval, Q1 - Q4 quartiles, T1 - T3 tertiles

*P-value from test for trend across ordered categories

**Fig. 3:**
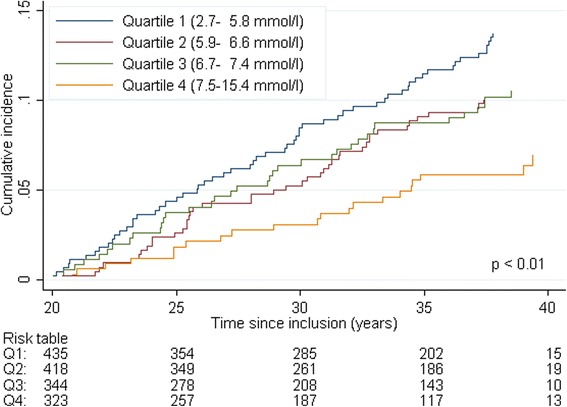
Cumulative incidence of prostate cancer by quartiles of cholesterol restricted to men with 20 years or more follow-up time, *n* = 1520. The number of prostate cancer diagnosis in each quartile of cholesterol was 60 (quartile 1), 42 (quartile 2), 36 (quartile 3) and 21 (quartile 4). *P*-value achieved from the Pepe and Mori test comparing the cumulative incidence of cholesterol quartile 1 versus quartile 4

Results from sensitivity analyses restricted to men who were still alive and free from prostate cancer at 20 years of follow-up (Fig. 3) show similar results as those obtained for full-time follow-up (Fig. 2 and Table 3).

**Fig. 4:**
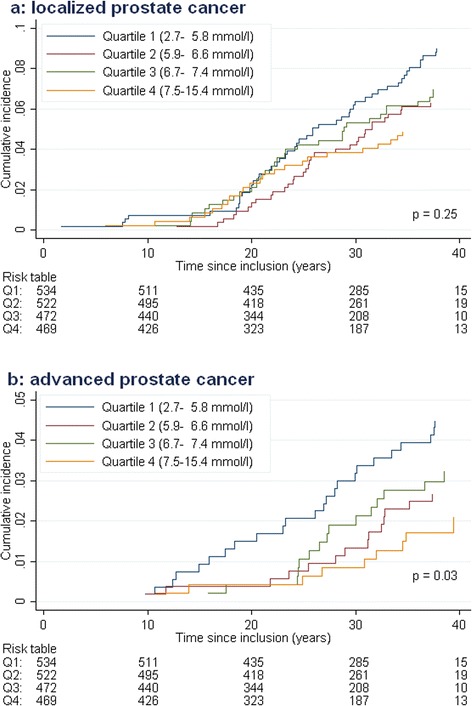
Cumulative incidence of **a** localized and **b** advanced stage prostate cancer by quartiles of cholesterol. The number of localized prostate cancer cases in quartiles of cholesterol was 48, 33, 33, 23, respectively, and the number of advanced prostate cancer cases was 24, 14, 15, 9, accordingly. *P*-values achieved from the Pepe and Mori test comparing the cumulative incidence of cholesterol quartile 1 versus quartile 4. Fully adjusted Fine and Gray competing risk model is presented in Additional file 1: Table S2A

Localized stage prostate cancer was not associated with cholesterol levels (Fig. 4a), while results for advanced stage cancer (Fig. 4b) confirmed the overall result of increased risk of prostate cancer in the first quartile as compared to the fourth quartile of cholesterol. However, the fully adjusted Fine and Gray competing risk models demonstrated an association between cholesterol and prostate cancer incidence for both stages (Additional file 1: Table S2A), and advanced stage cancer and prostate cancer-specific death in combined (Additional file 1: Table S2B).

Among men diagnosed with prostate cancer, 81 (38 %) died of the disease with mean age at death 79.5 years (Additional file 1: Table S3). No associations were observed between cholesterol (and the other covariates) and risk of prostate cancer-specific death (Additional file 1: Table S4 and Figure S1).

## Discussion

In this cohort of initially healthy middle-aged men, we found a negative association between baseline cholesterol levels and the incidence of prostate cancer. The finding is in contrast to the majority of studies in this field, which have pointed to a positive relationship between cholesterol and incidences of overall prostate cancer [8–12] or advanced stage prostate cancer [9, 11–14].

We consider it important to report this contradiction for several reasons. Firstly, we have taken risk of competing events into account when analyzing the association between cholesterol level and prostate cancer. Since cholesterol is a well-known risk factor of cardiovascular disease and mortality [20], statistical procedures in which deaths are censored may lead to biased conclusions [34]. Among the above-cited studies that have used a prospective longitudinal design [9, 10, 12–14], none included competing risk analyses. Among prospective studies that have found no associations between cholesterol and prostate cancer risk [4–7], only the study by van Hemelrijck et al. [4] has performed competing risk analyses.

Secondly, the long follow-up in our study made it possible to conduct an additional analysis in which we excluded all deaths and prostate cancer cases occurring during the first 20 years of observation. Results similar to those obtained without excluding data reduce the likelihood of reversed causality due to the possibility that early stages of prostate cancer may affect baseline levels of cholesterol.

Moreover, we observed that both high physical fitness and low BMI were independently associated with the risk of prostate cancer. Based on the most authoritative scientific research worldwide, the relationship between physical activity and prostate cancer is inconclusive [35]. However, the majority of these studies are based on self-reported levels of physical activity and, as far as we know, no studies have reported the relationship between measured physical fitness and risk of prostate cancer in a similar way to that presented here. Similarly, the relationship between overweight or obesity and risk of prostate cancer is inconclusive, although the evidence is considered to be strong for a relationship between high BMI and risk of advanced stage disease [35].

It is important to consider the possibility that screening procedures for prostate cancer may have influenced our findings [18]. Testing for Prostate Specific Antigen (PSA) has since the early 1990s strongly influenced the incidence rates of prostate cancer and especially increased the proportion of non-aggressive tumors.

Individuals who have an increased awareness of health, or belong to a social environment with more attention to disease prevention, may have lower cholesterol levels and simultaneously be more likely to attend tests for prostate cancer. This may explain the association between lower cholesterol levels and higher prostate cancer incidence. However, this hypothesis would primarily apply to the relationship between cholesterol and localized stage cancer and be less relevant for advanced stage cancer. In the present study, the association between cholesterol levels and incidence of localized stage prostate cancer was weaker than the association between cholesterol levels and advanced stage cancer, which reduces the likelihood of a biased selection to prostate cancer screening tests as a possible explanation of the association. Furthermore, testing for PSA received no application of importance in Norway until the mid-1990s. The proportion of person-years in the study cohort which was in the target group for PSA testing (≤70 years) after 1995, was less than 6 %. Thus, PSA testing had minimal impact on the findings in the present study.

Prospective studies assessing the association between baseline cholesterol and prostate cancer incidence will generally be vulnerable to substantial changes in lipid metabolism during follow-up. Some participants might have developed hypercholesterolemia during the observation period. Further, some participants with high cholesterol levels might have received medical treatment. In Norway, statins have been available for prescription use since the early 1990-ies, and the cholesterol-lowering effect of statins may have influenced our results. However, changes in cholesterol levels can in general be seen as regression to the mean and will largely serve to reduce the strength of the estimated associations.

Another limitation is lack of data on cellular atypia or glandular architecture [36] which may serve as powerful prognostic factors for prostate cancer. In the present study stage at diagnosis was restricted to two categories, localized cancer defined as invasive cancer without metastases, and advanced prostate cancer with infiltration into surrounding structures or metastases.

Strengths of our study include that it is among the longest and most complete follow-up studies of initially working and healthy middle-aged men. Similar cancer incidences as in the general population indicate high external validity for Caucasian populations in high income countries. Cholesterol levels were determined by standardized methods. Physical fitness was measured by an exercise test, as opposed to self-reports of physical activity typically used in epidemiological studies that may underestimate the association between physical activity and health outcomes [37]. By using physical examinations to measure height and weight we also eliminated a risk of error and response biases that is associated with self-reported height and weight [38].

## Conclusions

Low cholesterol, as well as high physical fitness and low BMI, may be associated with increased risk of prostate cancer. The relationship applies even to advanced stage cancer, which to a less extent is influenced by PSA testing. These findings are contrary to the advice about healthy lifestyle in the prevention of prostate cancer and may be important in the discussion on whether cholesterol affects the risk of prostate cancer.

## Additional file

Additional file 1: Table S1. Cox regression to evaluate the effect of selected covariates on prostate cancer incidence ignoring death as competing event (treated as censored), *n* = 1997. **Table S2A.** Cox and competing risk regression to evaluate the effect of selected covariates on (a) localized and (b) advanced stage prostate cancer incidence. *N* = 1997, whereof 137 localized and 62 advanced stage prostate cancer cases. **Table S2B.** Cox and competing risk regression to evaluate the effect of selected covariates on advanced stage prostate cancer incidence and prostate cancer-specific death in combined. *N* = 1997, whereof 105 events (62 advanced stage prostate cancer cases and 43 deaths of prostate cancer). **Table S3.** Number and age of death stratified by quartiles of cholesterol, Oslo Ischemia Study. **Table S4.** Competing risk regression to evaluate the effect of selected covariates on prostate cancer-specific death in men diagnosed with prostate cancer. 213 men were diagnosed with prostate cancer, whereof 81 died of prostate cancer (failure), 82 died of other causes (competing event), and 50 were alive at end of follow-up (censored). **Figure S1.** Cumulative incidence of prostate cancer death by quartiles of cholesterol in men diagnosed with prostate cancer, *n* = 213. Death of other causes was considered as competing events. P-value achieved from the Pepe and Mori test comparing the cumulative incidence of cholesterol quartile 1 versus quartile 4. (DOC 147 kb)

## Abbreviations

BMI: Body Mass Index (Body Weight/Height^2^)
PSA: Prostate Specific Antigen
SHR: Sub-Hazard Ratios
SIR: Standardized Incidence Rate
