# Supplementary material for: Cholesterol and prostate cancer risk: a long-term prospective cohort study
Source: BMC Cancer. 2016 Aug 17;16:643. doi: 10.1186/s12885-016-2691-5 (PMC4989293; doi:10.1186/s12885-016-2691-5)

Additional file 1

**Table S1**: Cox regression to evaluate the effect of selected covariates on prostate cancer incidence ignoring death as competing event (treated as censored), n=1997.

|  | Multivariate analysis | |
| --- | --- | --- |
|  | HR (95% CI) | *P-value* |
| Cholesterol (mmol/l) |  | 0.01* |
| Q1 (2.7 – 5.8) | 1.71 (1.14-2.58) | 0.01 |
| Q2 (5.9 – 6.6) | 1.13 (0.73-1.75) | 0.59 |
| Q3 (6.7 – 7.4) | 1.30 (0.84-2.02) | 0.24 |
| Q4 (7.5 – 15.4) | 1 |  |
| Age at inclusion (years) |  | <0.001* |
| <45 | 1 |  |
| 45-49 | 1.05 (0.73-1.52) | 0.79 |
| 50-54 | 1.68 (1.14-2.48) | 0.01 |
| 55+ | 2.49 (1.60-3.87) | <0.001 |
| Smoking |  |  |
| Never | 1 |  |
| Ever | 0.94 (0.70-1.26) | 0.66 |
| Physical fitness (kJ/kg) |  | 0.35* |
| T1 ( 21.1 – 118.6) | 1 |  |
| T2 (118.7 – 161.2) | 1.22 (0.84-1.76) | 0.30 |
| T3 (161.3 – 515.9) | 1.23 (0.84-1.80) | 0.28 |
| Body mass index (kg/m^2^) |  |  |
| <25 | 1 |  |
| ≥ 25 | 0.69 (0.51-0.93) | 0.01 |
| Systolic blood pressure (mmHg) |  | 0.09* |
| Q1 ( 88 – 118) | 1 |  |
| Q2 (120 – 128) | 0.99 (0.68-1.43) | 0.94 |
| Q3 (130 – 140) | 1.08 (0.73-1.58) | 0.71 |
| Q4 (142 – 220) | 1.43 (0.98-2.09) | 0.07 |

HR = hazard ratios; CI = confidence interval

Q1 – Q4 quartiles, T1 - T3 tertiles

*P-value for trend across ordered categories

**Table S2A**: Cox and competing risk regression to evaluate the effect of selected covariates on (a) localized and (b) advanced stage prostate cancer incidence. N=1997, whereof 137 localized and 62 advanced stage prostate cancer cases.

|  | Localized stage prostate cancer | | | | Advanced stage prostate cancer | | | |
| --- | --- | --- | --- | --- | --- | --- | --- | --- |
|  | Multivariate cox-analysis | | Multivariate competing risk analysis | | Multivariate cox-analysis | | Multivariate competing risk analysis | |
|  | HR (95% CI) | *P-value* | SHR (95% CI) | *P-value* | HR (95% CI) | *P-value* | SHR (95% CI) | *P-value* |
| Cholesterol (mmol/l) |  | 0.17* |  | 0.07* |  | 0.12* |  | 0.05* |
| Q1 (2.7 – 5.8) | 1.50 (0.90-2.49) | 0.12 | 1.70 (1.02-2.81) | 0.04 | 2.04 (0.93-4.44) | 0.07 | 2.30 (1.05-5.04) | 0.04 |
| Q2 (5.9 – 6.6) | 1.07 (0.63-1.84) | 0.79 | 1.22 (0.72-2.09) | 0.46 | 1.22 (0.52-2.84) | 0.64 | 1.34 (0.57-3.16) | 0.51 |
| Q3 (6.7 – 7.4) | 1.32 (0.77-2.26) | 0.31 | 1.37 (0.80-2.35) | 0.24 | 1.58 (0.69-3.62) | 0.28 | 1.62 (0.70-3.74) | 0.26 |
| Q4 (7.5 – 15.4) | 1 |  | 1 |  | 1 |  | 1 |  |
| Age at inclusion (years) |  | 0.03* |  | 0.65* |  | <0.001* |  | 0.20* |
| <45 | 1 |  | 1 |  | 1 |  | 1 |  |
| 45-49 | 0.80 (0.51-1.25) | 0.33 | 0.70 (0.45-1.09) | 0.12 | 2.10 (0.98-4.53) | 0.06 | 1.81 (0.85-3.89) | 0.13 |
| 50-54 | 1.06 (0.65-1.73) | 0.82 | 0.70 (0.42-1.15) | 0.16 | 4.20 (1.93-9.12) | <0.001 | 2.49 (1.12-5.52) | 0.03 |
| 55+ | 1.95 (1.15-3.30) | 0.01 | 0.93 (0.56-1.54) | 0.79 | 3.63 (1.38-9.55) | <0.01 | 1.42 (0.53-3.79) | 0.48 |
| Smoking |  |  |  |  |  |  |  |  |
| Never | 1 |  | 1 |  | 1 |  | 1 |  |
| Ever | 1.00 (0.69-1.45) | 0.997 | 0.87 (0.60-1.26) | 0.45 | 1.06 (0.61-1.87) | 0.83 | 0.89 (0.50-1.56) | 0.68 |
| Physical fitness (kJ/kg) |  | 0.48* |  | 0.07* |  | 0.74* |  | 0.20* |
| T1 ( 21.1 – 118.6) | 1 |  | 1 |  | 1 |  | 1 |  |
| T2 (118.7 – 161.2) | 1.16 (0.73-1.84) | 0.53 | 1.31 (0.83-2.08) | 0.25 | 1.29 (0.66-2.51) | 0.46 | 1.56 (0.77-3.16) | 0.21 |
| T3 (161.3 – 515.9) | 1.20 (0.75-1.92) | 0.45 | 1.55 (0.97-2.49) | 0.07 | 1.17 (0.58-2.37) | 0.65 | 1.67 (0.79-3.50) | 0.18 |
| Body mass index (kg/m^2^) |  |  |  |  |  |  |  |  |
| <25 | 1 |  | 1 |  | 1 |  | 1 |  |
| ≥ 25 | 0.69 (0.47-1.00) | 0.047 | 0.70 (0.49-1.00) | 0.052 | 0.65 (0.37-1.14) | 0.13 | 0.66 (0.37-1.16) | 0.15 |
| Systolic blood pressure (mmHg) |  | 0.10* |  | 0.39* |  | 0.45* |  | 0.97* |
| Q1 ( 88 – 118) | 1 |  | 1 |  | 1 |  | 1 |  |
| Q2 (120 – 128) | 1.01 (0.63-1.61) | 0.98 | 0.93 (0.58-1.48) | 0.75 | 1.11 (0.58-2.13) | 0.76 | 0.97 (0.50-1.87) | 0.93 |
| Q3 (130 – 140) | 1.32 (0.83-2.10) | 0.24 | 1.20 (0.76-1.92) | 0.44 | 0.65 (0.28-1.48) | 0.30 | 0.55 (0.25-1.24) | 0.15 |
| Q4 (142 – 220) | 1.41 (0.87-2.30) | 0.16 | 1.15 (0.71-1.86) | 0.58 | 1.60 (0.82-3.13) | 0.17 | 1.20 (0.63-2.28) | 0.59 |

HR = hazard ratios; SHR = sub-distribution hazard ratios; CI = confidence interval; Q1 – Q4 quartiles, T1 - T3 tertiles

* P-value for trend across ordered categories

**Table S2B**: Cox and competing risk regression to evaluate the effect of selected covariates on advanced stage prostate cancer incidence and prostate cancer-specific death in combined. N=1997, whereof 105 events (62 advanced stage prostate cancer cases and 43 deaths of prostate cancer).

|  | Multivariate cox-analysis | | Multivariate competing risk analysis | |
| --- | --- | --- | --- | --- |
|  | HR (95% CI) | *P-value* | SHR (95% CI) | *P-value* |
| Cholesterol (mmol/l) |  | 0.01* |  | 0.003* |
| Q1 (2.7 – 5.8) | 2.33 (1.26-4.31) | 0.007 | 2.67 (1.43-5.01) | 0.002 |
| Q2 (5.9 – 6.6) | 1.41 (0.73-2.73) | 0.30 | 1.57 (0.81-3.06) | 0.18 |
| Q3 (6.7 – 7.4) | 1.64 (0.84-3.17) | 0.15 | 1.68 (0.86-3.27) | 0.13 |
| Q4 (7.5 – 15.4) | 1 |  | 1 |  |
| Age at inclusion (years) |  | <0.001* |  | 0.06* |
| <45 | 1 |  | 1 |  |
| 45-49 | 1.86 (1.04-3.33) | 0.04 | 1.64 (0.92-2.92) | 0.09 |
| 50-54 | 3.52 (1.95-6.35) | <0.001 | 2.30 (1.25-4.22) | 0.007 |
| 55+ | 3.48 (1.72-7.03) | 0.001 | 1.60 (0.79-3.22) | 0.19 |
| Smoking |  |  |  |  |
| Never | 1 |  | 1 |  |
| Ever | 0.92 (0.60-1.40) | 0.70 | 0.77 (0.50-1.17) | 0.22 |
| Physical fitness (kJ/kg) |  | 0.64* |  | 0.11* |
| T1 ( 21.1 – 118.6) | 1 |  | 1 |  |
| T2 (118.7 – 161.2) | 1.20 (0.72-1.99) | 0.49 | 1.42 (0.85-2.36) | 0.18 |
| T3 (161.3 – 515.9) | 1.16 (0.68-1.98) | 0.58 | 1.58 (0.92-2.71) | 0.10 |
| Body mass index (kg/m^2^) |  |  |  |  |
| <25 | 1 |  | 1 |  |
| ≥ 25 | 0.68 (0.45-1.05) | 0.08 | 0.69 (0.45-1.05) | 0.08 |
| Systolic blood pressure (mmHg) |  | 0.27* |  | 0.85* |
| Q1 ( 88 – 118) | 1 |  | 1 |  |
| Q2 (120 – 128) | 1.16 (0.69-1.93) | 0.58 | 1.04 (0.62-1.74) | 0.88 |
| Q3 (130 – 140) | 0.90 (0.50-1.61) | 0.72 | 0.79 (0.44-1.40) | 0.42 |
| Q4 (142 – 220) | 1.53 (0.90-2.60) | 0.12 | 1.18 (0.70-1.97) | 0.54 |

HR = hazard ratios; SHR = sub-distribution hazard ratios; CI = confidence interval;

Q1 – Q4 quartiles, T1 - T3 tertiles

* P-value for trend across ordered categories

**Table S3**: Number and age of death stratified by quartiles of cholesterol, Oslo Ischemia Study.

|  |  | Cholesterol (mmol/l) | | | | |
| --- | --- | --- | --- | --- | --- | --- |
|  |  | Total | Q1  (2.7- 5.8) | Q2  (5.9- 6.6) | Q3  (6.7- 7.4) | Q4  (7.5-15.4) |
| Status at end of follow-up,  n (%) | Death overall | 1511 (75.7) | 365 (68.4) | 375 (71.8) | 371 (78.6) | 400 (85.3) |
|  | - Prostate cancer- specific death | 81 (4.1) | 34 (6.4) | 17 (3.3) | 19 (4.0) | 11 (2.3) |
|  | - Death of other causes | 1430 (71.6) | 331 (62.0) | 358 (68.6) | 352 (74.6) | 389 (82.9) |
| Age at death,  mean (95% CI) years | Death overall | 76.1  (75.6-76.6) | 76.7  (75.7-77.6) | 76.3  (75.4-77.2) | 75.9  (74.9-76.8) | 75.7  (74.7-76.6) |
|  | - Prostate cancer- specific death | 79.5  (78.1-81.0) | 79.9  (77.8-82.0) | 78.9  (75.7-82.2) | 79.5  (76.3-82.7) | 79.4  (74.6-84.1) |
|  | - Death of other causes | 75.9  (75.4-76.4) | 76.3  (75.3-77.3) | 76.2  (75.3-77.1) | 75.7  (74.7-76.7) | 75.6  (74.6-76.5) |

CI = confidence interval; Q1 – Q4 quartiles

**Table S4**: Competing risk regression to evaluate the effect of selected covariates on prostate cancer-specific death in men diagnosed with prostate cancer. 213 men were diagnosed with prostate cancer, whereof 81 died of prostate cancer (failure), 82 died of other causes (competing event), and 50 were alive at end of follow-up (censored).

|  | Multivariate competing  risk analysis | |
| --- | --- | --- |
|  | SHR (95% CI) | *P-value* |
| Cholesterol (mmol/l) |  | 0.18* |
| Q1 (2.7 – 5.8) | 1.65 (0.78-3.48) | 0.19 |
| Q2 (5.9 – 6.6) | 1.20 (0.53-2.71) | 0.66 |
| Q3 (6.7 – 7.4) | 1.51 (0.68-3.35) | 0.31 |
| Q4 (7.5 – 15.4) | 1 |  |
| Age at diagnosis (years) |  | 0.40* |
| <65 | 1 |  |
| 65-74 | 1.21 (0.55-2.65) | 0.63 |
| 75+ | 1.43 (0.65-3.13) | 0.37 |
| Smoking |  |  |
| Never | 1 |  |
| Ever | 0.92 (0.56-1.51) | 0.73 |
| Physical fitness (kJ/kg) |  | 0.84* |
| T1 ( 21.1 – 118.6) | 1 |  |
| T2 (118.7 – 161.2) | 0.96 (0.55-1.68) | 0.88 |
| T3 (161.3 – 515.9) | 0.96 (0.54-1.74) | 0.90 |
| Body mass index (kg/m^2^) |  |  |
| <25 | 1 |  |
| ≥ 25 | 1.44 (0.87-2.38) | 0.16 |
| Systolic blood pressure (mmHg) |  | 0.54* |
| Q1 ( 88 – 118) | 1 |  |
| Q2 (120 – 128) | 1.13 (0.62-2.07) | 0.68 |
| Q3 (130 – 140) | 0.93 (0.47-1.83) | 0.83 |
| Q4 (142 – 220) | 1.26 (0.69-2.30) | 0.44 |

SHR = sub-distribution hazard ratios; CI = confidence interval

Q1 – Q4 quartiles, T1 - T3 tertiles

* P-value for trend across ordered categories

**Figure S1**: Cumulative incidence of prostate cancer death by quartiles of cholesterol in men diagnosed with prostate cancer, n=213. Death of other causes was considered as competing events. P-value achieved from the Pepe and Mori test comparing the cumulative incidence of cholesterol quartile 1 versus quartile 4.


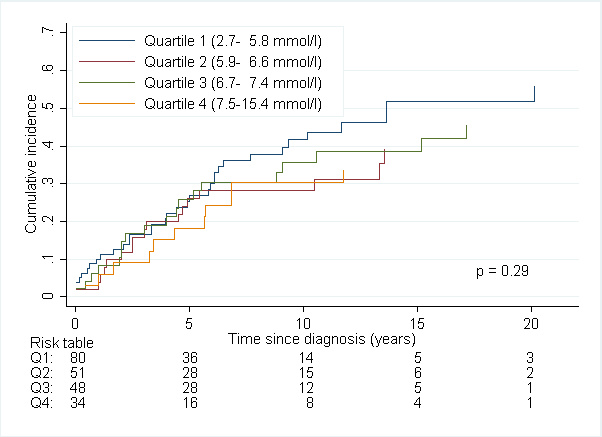

Supplement: Additional file 1: Table S1. — Cox regression to evaluate the effect of selected covariates on prostate cancer incidence ignoring death as competing event (treated as censored), n = 1997. Table S2A. Cox and competing risk regression to evaluate the effect of selected covariates on (a) localized and (b) advanced stage prostate cancer incidence. N = 1997, whereof 137 localized and 62 advanced stage prostate cancer cases. Table S2B. Cox and competing risk regression to evaluate the effect of selected covariates on advanced stage prostate cancer incidence and prostate cancer-specific death in combined. N = 1997, whereof 105 events (62 advanced stage prostate cancer cases and 43 deaths of prostate cancer). Table S3. Number and age of death stratified by quartiles of cholesterol, Oslo Ischemia Study. Table S4. Competing risk regression to evaluate the effect of selected covariates on prostate cancer-specific death in men diagnosed with prostate cancer. 213 men were diagnosed with prostate cancer, whereof 81 died of prostate cancer (failure), 82 died of other causes (competing event), and 50 were alive at end of follow-up (censored). Figure S1. Cumulative incidence of prostate cancer death by quartiles of cholesterol in men diagnosed with prostate cancer, n = 213. Death of other causes was considered as competing events. P-value achieved from the Pepe and Mori test comparing the cumulative incidence of cholesterol quartile 1 versus quartile 4. (DOC 147 kb) [file 12885_2016_2691_MOESM1_ESM.doc]
